# Alternative Protein Sources vs. GM Soybean Meal as Feedstuff for Pigs—Meat Quality and Health-Promoting Indicators

**DOI:** 10.3390/ani11010177

**Published:** 2021-01-13

**Authors:** Marcin Sońta, Anna Rekiel, Justyna Więcek, Martyna Batorska, Kamila Puppel

**Affiliations:** Department of Animal Breeding, Institute of Animal Sciences, Warsaw University of Life Sciences—SGGW, Ciszewskiego 8, 02-786 Warsaw, Poland; anna_rekiel@sggw.edu.pl (A.R.); justyna_wiecek@sggw.edu.pl (J.W.); martyna_batorska@sggw.edu.pl (M.B.); kamila_puppel@sggw.edu.pl (K.P.)

**Keywords:** fatteners, alternative protein sources, sustainable feed, meat quality, health-promoting indices

## Abstract

**Simple Summary:**

In its slogan “International year of pulses” coined for 2016, the WHO has indicated trends in activities for future generations, these being “Nutritious seeds for a sustainable future”. Alternative sources of vegetable protein can be used in the conditions of sustainable development to produce high-quality animal products, including pork. One of the goals of the European Green Deal is to produce protein feedstuff in Europe and become independent from imported American soybeans. This study aimed to partially replace genetically-modified soybean meal (SBM GM) with alternative protein sources (pea seeds and rapeseeds meal—RSM) in feed mixtures for growing-finishing pigs and to determine its impact on meat quality and health-promoting indices. The SBM GM was the only protein source in feed mixtures for control pigs. In feed mixtures for experimental animals, it was replaced with increasing doses of pea seeds, i.e., 5.0, 10.0, 15.0, and 17.5% in groups E1, E2, E3, and E4, respectively. The doses were the same in both fattening stages. The feed mixtures were iso-energetic and iso-protein. After completed fattening, meat was determined for quality attributes, fatty acid profile, and values of health-promoting indices beneficial from the dietetic perspective. Values of the analyzed quality attributes of pork justify using alternative protein sources as partial SBM GM replacers in the feeding of growing pigs.

**Abstract:**

This study aimed to explain the possibility of partial replacement of genetically-modified soybean meal (SBM GM) with pea seeds and rapeseed meal (RSM) in complete feed mixtures for growing-finishing pigs and to determine its impact on meat quality and health-promoting indices. The pigs (n = 50) were randomly divided into five groups, 10 animals each (gilts and barrows, 1:1, 3-breed: ♀ (landrace × yorkshire) × ♂ duroc), including the control group (C) and four experimental groups (E1, E2, E3, E4), and fed complete feed mixtures. The SBM GM was the only protein source in feed mixtures for control pigs. In feed mixtures for E1–E4 groups, it was partially replaced with pea seed doses of 5.0%, 10.0%, 15.0%, and 17.5% in groups E1, E2, E3, and E4, respectively. The feed mixtures were iso-energetic and iso-protein. After completed fattening, the animals were slaughtered. *M*. *longissimus lumborum* was sampled for analyses of the chemical and physical traits. The fatty acid profile determined in intramuscular fat (IMF) was used to compute the values of the health-promoting indices. The chemical and physical characteristics of meat were comparable in all groups. The study showed a dietetically-beneficial decrease in the values of atherogenicity index (AI), thrombogenicity index (TI), and saturation (S/P) in the meat of the experimental pigs vs. control group. The values of most of the analyzed quality attributes of pork justify using alternative protein sources as partial SBM GM replacers in diets for growing-finishing pigs in sustainable animal production.

## 1. Introduction

In feed mixtures for monogastric animals (pigs and poultry), genetically-modified soybean meal (SBM GM) is the main protein source. Results of research carried out in the last two decades have not confirmed any adverse effects of first-generation GM plant feedstuffs on the health of animals [1] nor on the food of animal origin [2]. Despite that, most of the consumers do not perceive foods containing or produced from GM as safe. To decrease SBM GM content in feedstuffs, alternative sources of protein have been extensively searched for in recent years. Non-processed and/or processed legume seeds, as well as insect proteins, potato proteins, or duckweed protein, have been used to this end [3,4]. Today, however, it seems that the greatest potential is offered by legume seeds [5]. The local production of peas entails lower plant and seed production costs compared to those incurred for purchasing imported GM soybean meal.

Degola and Jonkus [6] as well as Hanczakowska et al. [7] showed no adverse effects of using legume seeds in pig feeding on meat quality. In contrast, Hanczakowska and Świątkiewicz [8] and Sirtori et al. [9] showed various effects of feed mixtures containing legume seeds on the quality attributes of pork. Considering results reported by many authors [8,9,10,11,12,13,14] from studies into the usability of legume seeds in fatteners feeding and into their inexplicit effect on pork quality continued extensive research in this respect seems entirely justified. This is also supported by findings presented in a review work by Rungruangmaitree and Jiraungkoorskul [15] who emphasized the health-promoting properties of legume seeds and confirmed antibacterial, antidiabetic, antimycotic, anti-inflammatory, anti-hypercholesterolemic, antioxidative, and anticarcinogenic activities of pea seeds.

Red meat is a source of essential and very valuable nutrients for humans, including full-value protein (amino acids), B-group vitamins as well as Fe, Se, and Zn. They allow the body to grow and develop, build cells, and heal wounds. They also determine immunity, reduce susceptibility to stress, and stimulate the development of the nervous systems and thought processes in the brain. They perform essential metabolic functions, which justifies the consumption of meat, including pork [16,17]. Subcutaneous fat as well as intramuscular and intermuscular fat in pigs are an integral part of the meat. Their quality is important for consumers in the sensory and dietetic assessment. Beneficial modification of their composition is feasible via appropriate feeding methods.

This study aimed to determine the effect of the partial replacement of genetically-modified soybean meal with pea seeds and rapeseed meal in feed mixtures for growing-finishing pigs on the quality and health-promoting indices of meat.

## 2. Materials and Methods

According to Polish law and the EU Directive [18], the experiment did not require approval from the Local Ethical Committee because it was carried out under the production conditions of the family pig-producing farm. The farm uses available cereal components and a standard premix in pig nutrition. The experiment was conducted with growing pigs administered feed mixtures in which SBM-GM was partially replaced with pea seeds and rapeseed meal (RSM).

### 2.1. Animals

The experiment was performed with 3-breed Danish porkers: ♀ (landrace × yorkshire) × ♂ duroc (barrow to gilt ratio—1:1), in diets of which SBM GM was partially replaced with various doses of pea (*Pisum sativum*) seeds and RSM (Table 1). The animals (n = 50) were randomly divided into 5 groups (control group—C, and experimental groups—E1, E2, E3, E4), and placed in group pens, 10 animals each. Housing conditions were consistent with the Regulation of the Minister of Agriculture and Rural Development (Regulation of the Minister of Agriculture and Rural Development of 15 February 2010). There was 1 m^2^ of pen area per head. The floor type in the pens was a part slatted (1/3 of the surface of the pen) and a solid floor (2/3 of the surface of the pen). The animals were taken feed from automatic feeders and water from the bowl drinker (controlled ad libitum). The microclimate in the piggery throughout the fattening period was as follows: temperature 18–19 °C, and moisture about 70%. Two-stage fattening was performed (stage I—6 weeks, stage II—7 weeks), from 26.7 to 122.0 kg.

### 2.2. Raw Materials of Feed Mixtures

Pea seeds (Hubal variety) and rapeseed meal were determined for the chemical composition (Table 2) with the gas chromatography method at the Central Agroecological Laboratory of the Lublin University of Environmental Sciences, Lublin, Poland, according to the Laboratory’s procedures (CLA/PLC/20, CLA/PLC/34). The tannin content of pea seeds was determined with the Kuhl and Ebmeier method [19], and when expressed per catechin content reached 0.338 mg/g dry matter (d.m.). The content of oligosaccharides in pea seeds was determined with the gas chromatography method [20]. The total content of oligosaccharides was at 81.00 mg/g d.m. and that of raffinose-family oligosaccharides at 51.17 mg/g d.m.

### 2.3. Feed Mixtures

The 2-stage fattening was performed with complete feed mixtures (Table 3 and Table 4), which contained four ground grains. SBM GM served as the protein component in feed mixtures for the control groups, whereas various doses of pea seeds were used to partially replace the soybean meal in feed mixtures for animals from the experimental groups. Rapeseed meal and soybean oil were used additionally to balance the feed mixtures for pigs from groups E1–E4. The feed mixtures were iso-energetic and iso-protein [21,22].

The pigs were fed ad libitum and had free access to water.

### 2.4. Meat Analysis

After completed fattening, all animals were slaughtered in compliance with the procedures adopted at the slaughterhouse. After 24-h chilling at a temperature of +4 °C, samples of *M. longissimus lumborum*—*MLL* were collected from right cold half-carcasses for qualitative analyses.

Pork samples (ca. 0.5 kg) were determined for the contents of water, protein, fat, and collagen using a FoodScan^TM^Lab meat analyzer by Foos [23].

Color measurements were performed in the CIE L*a*b* system using a CR-400/410 chroma meter by Konica Minolta [24]. The procedure of color determination included sampling a slice of meat (ca. 2 cm thick) at 3 points (results obtained were averaged). Hue (b*/a*) and chromaticity (√(a*^2^ + b*^2^)) of meat sample color were calculated according to the formula provided by Mordenti et al. [25].

To determine the free drip (Drip loss), a ca. 300-g sample of meat was put into a polyethylene bag and cold-stored (+4 °C) for 24 h. Afterward, the liquid (the drip) was poured out and its volume was expressed in percent relative to sample weight [26].

Using the weighted portion of ground meat (300 mg each), analyses were conducted to determine the water holding capacity (WHC) according to the methodology described by Grau and Hamm [27], modified by Pohja and Ninivarra [28].

The fatty acid profile was determined in the samples of *MLL*. Meat fat extraction (IMF) was carried out according to Folch [22]. Fatty acid methylation was performed according to the trans-esterification method EN ISO 5509 [29]. Individual fatty acids were identified in crude fat using an Agilent 7890A GC (Agilent, Waldbronn, Germany) according to Puppel et al. [30]. Each peak was identified using pure methyl ester standards: FAME Mix RM-6, Lot LB 68242; Supelco 37 Comp. FAME Mix, Lot LB 68887; Methyl linoleate, Lot 094K1497; CLA Conjugated (9Z, 11E), Lot BCBV3726 (Supelco, Bellefonte, PA, USA). The following groups of fatty acids were determined: SFAs—C14:0, C16:0, C18:0; monounsaturated fatty acids (MUFAs)—C16:1, C18:1 *cis* 9, C18:1 *cis* 11; and polyunsaturated fatty acids (PUFAs): C18:2, C18:3, C20:3, C20:4, C22:5. The determined contents of individual fatty acids and groups of fatty acids allowed computing: atherogenicity index—AI, thrombogenicity index—TI, and S/P saturation according to Ulbricht and Southgate [31], using the following formulas.

AI = (4 × C14:0 + C16:0)/(MUFA + PUFA)

TI = (C14:0 + C16:0 + C18:0)/(0.5 × MUFA + 0.5 × *n6* PUFA + 3 × *n3* PUFA + *n3*/*n6* PUFA)

S/P = (C14:0 + C16:0 + C18:0)/(MUFA *cis* + PUFA)

In turn, the below-presented formulas were used to compute the ratio of hypocholesterolemic fatty acids (DFA) to hypercholesterolemic fatty acids (OFA) [32]:

DFA/OFA = (C18:1 + C18:2 + C18:3 + C20:4 + C20:5 + C22:6)/(C14:0 + C16:0)

### 2.5. Statistical Analysis

The results from experiments were subjected to statistical analysis using the IBM SPSS Statistics 25 package. Means and standard deviations were used to describe the data. Differences between the groups were assessed using linear regression models with the control group used as a reference. Overall, significance of the model was obtained using F statistics, whereas significance for each level of the grouping variable was obtained using *t* statistics. *p* < 0.05 was considered to denote statistically significant differences.

## 3. Results

The results of fattening and slaughter performance of the fatteners were very good and did not differ significantly among the feeding groups (Table 5).

The initial body weight of fatteners was similar in the groups and reached 26.7 ± 0.9 kg. The highest final body weight was reached by the pigs from group E2 (124.8 kg) and the lowest one by those from group E4 (116.5 kg). Daily body weight gains did not differ statistically among the groups, with the highest ones noted in group E2 (1113 g) and the lowest ones in group E4 (1022 g). The mean meatiness of fatteners from groups examined was at 60.2%. In the control group, the feed conversion ratio (FCR) was 2.52 kg/kg body weight gains, whereas in the experimental groups it was higher by 0.05–0.11 kg/kg body weight gain.

### 3.1. Meat Quality

Statistically significant differences were determined in protein content of meat between fatteners from groups C and E1 (*p* < 0.010), E3 (*p* < 0.023), and E4 (*p* < 0.025). In contrast, the contents of collagen in meat were similar in all groups. Negligible differences were observed in fat content, with the greatest ones observed between group E4 and group C (by 0.9 percentage point).

No statistically significant differences were found between the fatteners from group C and groups E1–E4 in meat color components as well as in color hue and chromaticity (Table 6). The smallest WHC was determined in the *MLL* of the fatteners from group C and the highest one in the *MLL* of the pigs from group E2 (difference of 1.9 cm^2^/g).

### 3.2. Fatty Acid Profile and Health-Promoting Indices of Meat

The use of experimental diets has contributed to a reduction in the total content of saturated fatty acids (SFA) in the meat of fatteners from groups E1, E3, and E4 (Table 7). The lowest SFA content was determined in the meat of E4 fatteners—34.83 g/100g fat (*p* < 0.001). The reduction was by 13.21% compared to the control group (C). In groups E3 and E4, analyses have shown a decrease (*p* < 0.001) in contents of C14:0, C16:0, and C18:0 fatty acids compared to group C. The lowest contents of these acids were determined in group E; they were lower by 16.11%, 11.85%, and 17.16% than in group C. Meat from experimental fatteners was characterized by an increased sum of PUFA compared to the control group. The highest PUFA content was found in the meat fat of fatteners from group E; it was higher by 60.41% (*p* < 0.001) compared to fatteners from group C. In groups E1–E4, a decrease was noted in the values of AI, TI, and S/P indices compared to the control group (Table 7). In group E4, their values were lower by 20.99% (AI), 19.42% (TI), and 21.92% (S/P) compared to group C. The DFA:OFA ratio increased in the experimental groups (E1–E4), and its highest value (*p* < 0.001) was noted in group E4 (with a difference between E4 and C reaching 26.06%).

## 4. Discussion

### 4.1. Chemical and Physical Characteristics of Meat

Pea has been used in pig feeding in both scientific experiments and production as a partial or complete replacer of soybean meal GM. It has served as a sole or one of the few plant-derived protein components in complete feed mixtures [6,8,9,33,34,35].

The present study demonstrates that using plant protein sources in feed mixtures for fatteners as SGM GM replacers had no effect on the chemical composition and physical traits of their meat, except for protein content, the higher content of which was determined in E1–E4 groups vs. C group. Similar conclusions were reported by Gatta et al. [34] and Sirtori et al. [9], while such a change was not observed in studies conducted by Chrenková et al. [33] and Degola and Jonkus [6]. Results from the present study regarding the chemical and physical characteristics of meat confirm data reported earlier by Zralý et al. [36] and Hanczakowska et al. [37]. The optimal content of fat (IMF) in pork meat should be from 2% to 3%. In our study, it was higher, which improved meat juiciness and tenderness. Our study results indicate that meat of fatteners administered feed mixtures with plant protein source inclusion may be found of good quality.

### 4.2. Fatty Acids and Health-Promoting Indices

The fatty acid composition of animal products, like eggs, milk, and meat, is a reflection of both the biosynthesis of fatty acids and the composition of fatty acid taken with food. However, this relationship is stronger in monogastric animals (pigs) than in the ruminants, in which functional fatty acids are hydrogenated in the rumen [38].

In the pig, the privileged site of de novo lipid synthesis is the adipose tissue. Studies have shown that SFA intake is associated with an increased risk of coronary heart disease [39]. The C12:0, C14:0, and C16:0 FAs have all been found to raise low-density lipoprotein cholesterol. Thus, the pursuit of reducing SFA level in meat should be one of the primary research goals of studies aimed to improve dietetic and health-promoting meat properties. In groups E3 and E4, analyses have shown a decrease in contents of C14:0, C16:0, and C18:0 fatty acids compared to group C, which indicates a reduction in the degree of saturation, and the positive effect of the applied supplementation. In the experiment performed by Sirtori et al. [9], the inclusion of pea seeds (31%) in a diet for growing pigs caused an increase in SFA content compared to the control group. Opposite results were obtained in the present study wherein the content of this group of fatty acids decreased significantly, which is difficult to interpret and explain. Both mentioned experiments revealed a slight increase in MUFA content and a decrease in PUFA content in the experimental groups. Chrenková et al. [33] reported increased contents of SFA and MUFA and a decreased PUFA content in meat of fatteners receiving pea seeds (15%) in their diet. The analysis of results from the present study as well as the above-cited works [33,34,35], addressing the impact of pea seeds on meat quality, reveals significant differences regarding contents of fatty acid groups and ratios between them. This confirms the advisability of continued research and comparative analysis concerning the nutritionally important fatty acids in meat produced from pigs receiving feed mixtures containing protein from various plant sources, including diets with pea seeds inclusion.

After pea seeds (15%) and rapeseed meal (15%) inclusion to feed mixtures for growing pigs, Fiedorowicz-Szatkowska et al. [40] demonstrated decreased contents of SFA and PUFA and increased content of MUFA. A different relationship has been shown in the present study. The content of PUFA increased in the meat fat of fatteners from experimental groups compared to the control animals (Table 7), and its highest value was demonstrated in group E4—10.86 g/100g fat (*p* < 0.001), which was higher by 60.41% than in group C. The major PUFAs were C18:2 *n6* and C18:3 *n3*. They both have been found to prevent coronary heart disease [39]. With the decrease in backfat thickness, the content of C18:2 *n6* increases, as confirmed by the obtained results. In the present study, the content of C18:2 acid increased in the meat of experimental pigs compared to the control animals. Its highest value was noted in group E4—10.29 g/100g fat, which was higher by 84.74% compared to the control group. The increase in C18:2 *n6* is associated with a lower amount of endogenous lipids in the meat. This phenomenon has a negative impact on the fat tissue during precession (lowering the melting temperature) but also a positive one considering the health-promoting properties of meat (the content of essential fatty acids) [41].

The highest content of C18:1 *cis* 9 acid was demonstrated in the meat fat of group E2 fatteners; it was higher by 5.15% compared to group C. This FA has been reported to have anti-atherogenic protective effects on endothelial cells [42]. Increased C18:1 *cis* 9 concentration is beneficial for the antioxidant quality of the meat, which indicates a positive effect of the applied supplementation.

Experimental diets contributed to decreased values of the atherogenicity index (AI), thrombogenicity index (TI), saturation (S/P), and improved the dietetic quality of meat of the analyzed fatteners. In the present study, a beneficial decrease was noted in the values of these indices after pea seeds addition to feed mixtures, i.e., by ca. 21% (AI) and 19% (TI). However, the AI and TI values determined for the meat of fatteners (the present study) were higher than these reported for example the meat of wild boars [43], which is indicative of a better dietetic value of the game meat because of natural environment compared to pork obtained in the intensive fattening system. In the present study, the value of the saturation index (S/P) was comparable or lower in groups E1–E4 than in group C. This may suggest that the consumption of meat obtained from pigs fed vegetable-derived protein may contribute to a reduced risk of the development of ischemic heart disease. The ratio of hypocholesterolemic acids to hypercholesterolemic acids (DFA:OFA) in the present study was higher in the experimental groups than in the control group. The DFA:OFA value of meat products should approximate 2, which ensures that the fat is more appropriate for a man’s diet [44]. In our study, the DFA:OFA value was the lowest in group C (1.88) and higher in experimental groups (the highest in group E4—2.37). Its increased values determined in groups E1–E2 vs. C can be found beneficial from the dietetic point of view.

## 5. Conclusions

Results achieved in the conducted experiment enable concluding that feed mixtures containing pea seeds and rapeseed meal as SBM GM replacers had no unbeneficial effect on the chemical and physical characteristics of pork, which is indicative of the practical usability of pea seeds and rapeseed meal as a feedstuff material for growing-finishing pigs. The quality of pork obtained from the fatteners may be found good. The beneficial changes in contents of C18:2 and PUFAs justify the use of pea seeds and rapeseed meal in diets for fatteners. The improvement of the dietetic value of pork, expressed by the decreased values of AI, TI, and S/P, as well as a high dietetic quality of meat fat evidenced by DFA:OFA value (groups E1 and E2), confirm the advisability of using pea seeds and rapeseed meal of protein as partial replacers of GM soybean meal. The use of pea seeds and rapeseed meal in the feeding of fattening pigs is beneficial in terms of sustainable animal production.

## Figures and Tables

**Table 1:** Experiment design.

Content (%) of Raw Material in Feed Mixture		Groups			
	C	E1	E2	E3	E4
Soybean meal GM (SBM GM):					
I stage of fattening	13.0	9.7	8.3	6.4	2.0
II stage of fattening	10.5	6.6	4.8	3.0	-
Pea seeds—I and II stage of fattening	-	5.0	10.0	15.0	17.5
Rapeseed meal (RSM):					
I stage of fattening	-	2.5	2.5	2.5	7.8
II stage of fattening	-	2.5	2.5	2.2	6.0

**Table 2:** Chemical composition of pea seeds (Hubal variety) and rapeseed meal.

Components	Pea Seeds	Rapeseed Meal
%		
Dry matter	87.61	88.10
Crude protein	23.56	35.52
Crude fiber	3.63	3.56
Crude ash	2.51	7.25
Ether extract	0.82	1.12
NFE *	57.09	40.65
Amino acids, % protein		
Aspartic acid	25.10	27.90
Threonine	8.30	15.80
Serine	10.80	15.10
Glutamic acid	41.10	55.70
Proline	9.74	21.70
Glycine	9.01	19.20
Alanine	9.23	15.45
Cysteine	3.15	7.80
Valine	9.57	15.80
Methionine	2.26	6.90
Isoleucine	8.50	13.10
Leucine	15.20	22.40
Tyrosine	6.05	8.40
Phenylalanine	10.20	13.30
Histidine	5.34	8.80
Lysine	15.30	18.45
Arginine	18.70	20.50
Tryptophan	1.62	4.25
Fatty acids, %		
C14:0	0.32	1.52
C15:0	0.20	0.45
C16:0	9.82	7.45
C16:1 *n7*	0.02	-
C16:1 *n9*	0.16	1.80
C17:0	0.80	-
C17:1	0.05	-
C18:0	3.98	1.42
C18:1 *n9*	23.56	50.51
C18:1 *n7*	0.23	0.43
C18:2 *n6*	47.14	24.68
C18:3 *n3*	12.10	26.31
C20:0	0.69	0.51
C20:1 *n9*	0.47	0.82
C20:2	0.10	-
C20:3 *n6*	0.04	-
C22:0	0.30	2.90
C22:2	0.02	-

* NFE—nitrogen-free extract.

**Table 3:** Composition of feed mixtures at stage I of fattening (%).

Feed Raw Materials	Groups				
	C	E1	E2	E3	E4
Barley	35.0	30.0	25.0	15.0	5.0
Triticale	24.0	19.2	20.3	22.1	28.3
Wheat	20.0	25.0	25.0	30.0	30.0
Oats	5.0	5.0	5.0	5.0	5.0
Soybean meal GM	13.0	9.7	8.3	6.4	2.0
Rapeseed meal	–	2.5	2.5	2.5	7.8
Pea seeds	–	5.0	10.0	15.0	17.5
Soybean oil	–	0.6	0.9	1.0	1.4
Premix *	3.0	3.0	3.0	3.0	3.0
**Analyzed nutritional Value (%)**					
Dry matter	87.6	87.4	86.9	87.3	87.5
Crude protein	16.4	16.5	16.3	16.5	16.3
Ether extract	2.6	2.7	2.7	2.7	2.8
Crude fiber	3.9	3.9	4.0	4.1	4.1
Crude ash	3.9	4.0	3.9	4.1	3.9
**Calculated nutritional Value (%)**					
Metabolic energy (MJ/kg)	13.22	13.21	13.21	13.21	13.21
Lysine	1.05	1.06	1.09	1.10	1.10
Methionine + cysteine	0.64	0.64	0.64	0.63	0.67
Threonine	0.70	0.71	0.71	0.71	0.72
Tryptophan	0.20	0.19	0.19	0.19	0.19
Calcium	0.81	0.82	0.82	0.82	0.86
Phosphorus	0.54	0.55	0.55	0.54	0.58
Sodium	0.17	0.19	0.21	0.23	0.24

* 1 kg of premix contained: lysine—12.10%; methionine—2.65%; threonine—5.05%; tryptophan—0.25%; calcium—20.50%; phosphorus—1.80%; sodium—5.00%; iron—4000 mg; manganese—2400 mg; zinc—2600 mg; copper—800 mg; iodine—55.0 mg; selenium—13.50 mg; vitamin A—260,000 IU; vitamin D3—69,000 IU; vitamin E—4700 mg; vitamin K3—68 mg; vitamin B1—68 mg; vitamin B2—170 mg; vitamin B6—105 mg; vitamin B12—830 mcg; vitamin C—1000 mg; folic acid—27.00 mg; pantothenic acid—410 mg; niacinamide B3—690 mcg; biotin—3450 mg; choline chloride—10,000 mg; aroma, antioxidant: 1b (E320-BHA, E321-BHT, E324—ethoxyquin) 550 mg/kg; enzymes: 4a E-1 640 6—phytase (EC 3.1.3.2.6 n-5000 FTU/g) 17,500 FTU/kg, (E1600 endo 1,4-beta-xylanase, EC 3.2.1.8–22,000 VU/g; 425,000 VU/kg, endo 1,3 beta-glucanase EC 3.2.1.6–30,000 VU/g, 57,000 VU/kg); raw material composition: calcium carbonate, monocalcium phosphate, (monophosphate) sodium chloride 1.8.1.9, herbal mix 10 g/kg.

**Table 4:** Composition of feed mixtures at stage II of fattening (%).

Feed Raw Materials	Groups				
	C	E1	E2	E3	E4
Barley	35.0	25.0	15.0	10.0	8.2
Triticale	32.0	26.5	30.0	30.0	30.0
Wheat	10.0	21.6	24.7	30.0	30.0
Oats	10.0	10.0	10.0	6.9	5.0
Soybean meal GM	10.5	6.6	4.8	3.0	–
Rapeseed meal	–	2.5	2.5	2.2	6.0
Pea seeds	–	5.0	10.0	15.0	17.5
Soybean oil	–	0.3	0.5	0.4	0.8
Premix *	2.5	2.5	2.5	2.5	2.5
**Analyzed Nutritional Value (%)**					
Dry matter	86.8	86.4	87.0	86.7	86.1
Crude protein	15.3	15.4	15.5	15.2	15.1
Ether extract	2.5	2.6	2.6	2.6	2.7
Crude fiber	4.1	4.2	3.9	4.2	3.9
Crude ash	4.5	4.7	4.5	4.7	4.6
**Calculated Nutritional Value (%)**					
Metabolic energy (MJ/kg)	13.17	13.16	13.17	13.17	13.16
Lysine	0.94	0.94	0.95	0.96	0.97
Methionine + cysteine	0.60	0.61	0.61	0.60	0.61
Threonine	0.64	0.64	0.64	0.64	0.65
Tryptophan	0.18	0.18	0.18	0.17	0.17
Calcium	0.68	0.70	0.69	0.69	0.71
Phosphorus	0.51	0.51	0.51	0.50	0.52
Sodium	0.15	0.17	0.19	0.21	0.21

* Premix composition see: Table 3.

**Table 5:** Fattening and carcass results of pigs in experiment, ($\bar{x}$ ± SD).

Specification	Groups ^1^					*p*-Value
	C	E1	E2	E3	E4	
Initial body weight, kg	26.4 ± 1.1	27.1 ± 0.8	26.9 ± 0.9	26.5 ± 0.8	26.5 ± 1.0	0.336
Final body weight, kg	123.4 ± 9.6	123.0 ± 8.7	124.8 ± 7.5	122.1 ± 7.3	116.5 ± 9.7	0.255
Average daily body weight gain, g	1104 ± 119	1090 ± 97	1113 ± 87	1086 ± 80	1022 ± 108	0.294
Feed conversion rate, kg/kg b.w.	2.52	2.60	2.58	2.57	2.63	-
Meatiness, %	60.0 ± 0.8	60.6 ± 1.9	60.1 ± 2.3	60.4 ± 2.0	59.7 ± 2.4	0.854

^1^ C—soybean meal GM; E1—5.0% pea seeds; E2—10.0% pea seeds; E3—15.0% pea seeds; E4—17.5% pea seeds.

**Table 6:** Quality of meat from *Musculus longissimus lumborum* of fatteners, ($\bar{x}$ ± SD).

Specification	Groups ^1^					*p*-Value
	C	E1	E2	E3	E4	
Chemical composition (%):						
Water	70.9 ± 0.7	71.6 ± 0.5	71.4 ± 0.6	71.1 ± 0.6	71.6 ± 0.7	0.055
Protein	22.0 ^abc^ ± 0.3	22.4 ^a^ ± 0.3	22.2 ± 0.3	22.4 ^b^ ± 0.4	22.4 ^c^ ± 0.4	0.044
Fat	5.2 ± 0.8	4.6 ± 0.8	5.1 ± 0.5	4.8 ± 0.8	4.3 ± 0.9	0.075
Collagen	1.1 ± 0.3	1.1 ± 0.2	1.1 ± 0.2	1.2 ± 0.2	1.1 ± 0.3	0.863
Physical traits:						
Color components in the CIE scale:						
L*	50.6 ± 1.8	51.1 ±2.9	50.4±2.4	49.5±1.0	50.4±1.6	0.552
a*	7.2 ± 0.9	7.5 ± 1.0	7.9 ± 0.9	7.7 ± 1.0	8.2 ± 1.3	0.281
b*	3.2 ± 0.9	3.8 ± 0.8	3.9 ± 0.9	3.6 ± 1.1	3.9 ± 1.1	0.530
Hue	0.5 ± 0.1	0.5 ± 0.1	0.5 ± 0.1	0.5 ± 0.1	0.5 ± 0.1	0.699
Chromaticity	4.6 ± 0.3	4.8 ± 0.3	4.9 ± 0.3	4.7 ± 0.5	4.9 ± 0.4	0.287
Drip loss, %	4.6 ± 0.6	4.5 ± 0.9	4.5 ± 0.6	4.4 ± 0.9	5.1 ± 0.8	0.308
WHC ^2^, cm^2^/g	17.1 ± 2.4	15.6 ± 1.7	15.2 ± 3.6	16.6 ± 1.5	16.4 ± 1.9	0.349

^1^ C—soybean meal GM; E1—5.0% pea seeds; E2—10.0% pea seeds; E3—15.0% pea seeds; E4—17.5% pea seeds; ^2^ water holding capacity. a, a—values in the rows with the same letters differ significantly at *p* < 0.05.

**Table 7:** Fatty acid composition, contents of groups of fatty acids (g/100 g fat), and values of health-promoting indices, ($\bar{x}$ ± SD).

Specification	Groups ^1^					*p*-Value
	C	E1	E2	E3	E4	
C14:0	1.49 ^AB^ ± 0.16	1.50 ± 0.21	1.39 ± 0.10	1.26 ^A^ ± 0.06	1.25 ^B^ ± 0.09	0.001
C16:0	25.23 ^AB^ ± 1.29	25.77 ± 2.36	25.10 ± 1.17	22.53 ^A^ ± 1.01	22.24 ^B^ ± 1.39	0.001
C16:1	3.65 ± 0.33	3.66 ± 0.61	3.34 ± 0.25	3.41 ± 0.19	3.82 ± 0.35	0.072
C18:0	13.05 ^Aab^ ± 0.96	11.55 ^a^ ± 1.84	13.39 ± 1.28	11.59 ^b^ ± 0.60	10.81 ^A^ ± 0.96	0.001
C18:1 *cis9*	40.93 ± 4.22	42.29 ± 2.86	43.04 ± 2.10	42.74 ± 2.13	40.17 ± 2.77	0.204
C18:1 *cis11*	2.57 ^ABCa^ ± 0.70	1.53 ^A^ ± 0.41	1.66 ^B^ ± 0.56	3.03 ^a^ ± 0.32	3.70 ^C^ ± 0.55	0.001
C18:2	5.57 ^ABa^ ± 1.37	6.85 ^a^ ± 1.11	6.33 ± 0.38	8.65 ^A^ ± 0.66	10.29 ^B^ ± 1.37	0.001
C18:3	0.32 ^A^ ± 0.21	0.28 ± 0.17	0.21 ± 0.02	0.12 ^A^ ± 0.01	0.23 ± 0.15	0.041
C20:3	0.16 ^ABCa^ ± 0.09	0.11 ^a^ ± 0.03	0.08 ^A^ ± 0.03	0.07 ^B^ ± 0.02	0.03 ^C^ ± 0.01	0.001
C20:4	0.53 ^ABa^ ± 0.12	0.41 ^a^ ± 0.11	0.43 ± 0.23	0.31^A^ ± 0.04	0.23 ^B^ ± 0.10	0.001
C22:5	0.02 ^Aab^ ± 0.00	0.04 ^A^ ± 0.02	0.03 ^a^ ± 0.01	0.02 ± 0.01	0.03 ^b^ ± 0.01	0.001
SFAs	40.13 ^AB^ ± 1.98	39.10 ± 2.70	40.31 ± 1.99	35.87 ^A^ ± 1.62	34.83 ^B^ ± 2.24	0.001
MUFAs	48.08 ± 4.00	49.05 ± 2.91	49.21 ± 2.32	51.00 ± 2.42	48.98 ± 2.85	0.344
PUFAs	6.77 ^ABa^ ± 1.54	8.06 ^a^ ± 1.22	7.48 ± 0.48	9.35 ^A^ ± 0.72	10.86 ^B^ ± 1.43	0.001
AI	0.81 ^ABa^ ± 0.06	0.76 ^a^ ± 0.05	0.78 ± 0.03	0.65 ^A^ ± 0.03	0.64 ^B^ ± 0.02	0.001
TI	1.39 ^ABa ±^ 0.11	1.31 ^a^ ± 0.09	1.37 ± 0.06	1.15 ^A^ ± 0.05	1.12 ^B^ ± 0.05	0.001
DFA:OFA	1.88 ^AB^ ± 0.15	1.93 ± 0.17	1.98 ± 0.05	2.35 ^A^ ± 0.10	2.37 ^B^ ± 0.07	0.001
S/P	0.73 ^ABa^ ± 0.02	0.68 ^a^ ± 0.05	0.70 ± 0.03	0.59 ^A^ ± 0.03	0.57 ^B^ ± 0.02	0.001

^1^ C—soybean meal GM; E1—5.0% pea seeds; E2—10.0% pea seeds; E3—5.0% pea seeds; E4—17.5% pea seeds; AI—atherogenicity index; TI—thrombogenicity index; DFA:OFA—hypocholesterolemic to hypercholesterolemic fatty acids ratio; S/P—saturation; SFA—saturated fatty acids; MUFA—monounsaturated fatty acids; PUFA—polyunsaturated fatty acids; A, A—values in the rows with the same letters differ significantly at *p* < 0.01, a, a—values in the rows with the same letters differ significantly at *p* < 0.05

## Data Availability

The data presented in this study are available on request from the corresponding author. The data are not publicly available to preserve privacy of the data.
